# Digital approaches in post-COVID healthcare: a systematic review of technological innovations in disease management

**DOI:** 10.1093/biomethods/bpae070

**Published:** 2024-10-01

**Authors:** Pamela Mfouth Kemajou, Armand Mbanya, Yves Coppieters

**Affiliations:** School of Public Health, Centre for Research in Epidemiology, Biostatistics and Clinical Research, Université Libre de Bruxelles (ULB), Brussels, Belgium; Health of Population in Transition Research Group, University of Yaounde I, Yaounde, Cameroon; School of Public Health, Centre for Research in Epidemiology, Biostatistics and Clinical Research, Université Libre de Bruxelles (ULB), Brussels, Belgium

**Keywords:** post-COVID conditions, modern digital approaches, digital health technologies, smart healthcare systems, infodemic

## Abstract

Post-COVID conditions (PCC) emerged during the pandemic, prompting a rise in the use of Digital Health Technologies (DHTs) to manage lockdowns and hospital overcrowding. Real-time tracking and information analyses were crucial to strengthening the global research response. This study aims to map the use of modern digital approaches in estimating the prevalence, predicting, diagnosing, treating, monitoring, and prognosis of PCC. This review was conducted by searching PubMed and Scopus databases for keywords and synonyms related to DHTs, Smart Healthcare Systems, and PCC based on the World Health Organization definition. Articles published from 1 January 2020 to 21 May 2024 were screened for eligibility based on predefined inclusion criteria, and the PRISMA framework was used to report the findings from the retained studies. Our search identified 377 studies, but we retained 23 studies that used DHTs, artificial intelligence (AI), and infodemiology to diagnose, estimate prevalence, predict, treat, and monitor PCC. Notably, a few interventions used infodemics to identify the clinical presentations of the disease, while most utilized Electronic Health Records and AI tools to estimate diagnosis and prevalence. However, we found that AI tools were scarcely used for monitoring symptoms, and studies involving SHS were non-existent in low- and middle-income countries (LMICs). These findings show several DHTs used in healthcare, but there is an urgent need for further research in SHS for complex health conditions, particularly in LMICs. Enhancing DHTs and integrating AI and infodemiology provide promising avenues for managing epidemics and related complications, such as PCC.

## Introduction

During the COVID-19 pandemic and beyond, post-COVID conditions (PCC), also called long COVID, have presented a significant public health challenge. In this review, PCC is defined as the continuation or development of new symptoms 3 months after the initial SARS-CoV-2 infection, lasting at least 2 months with no other explanation. These symptoms might persist from their initial illness or develop after their recovery. They can come and go or relapse over time. This 3-month period allows healthcare providers to rule out the usual recovery period from an acute illness [1].

The traditional healthcare service delivery model, which is mainly patient-centred care, was overburdened due to the rapid spread of COVID-19 [2]. This necessitated swift and effective solutions for disease diagnosis, management, and treatment. As a result, timely and effective solutions were needed on how to diagnose and manage the disease and treatment. Such solutions were digital health platforms, telemedicine, and e-health technologies. They played an important role in improving the diagnosis, consultation, and treatment of patients by providing alternative means to visit the hospital, especially when the countries were under lockdown. However, they could not be validated and approved because of a lack of standardized regulations and guidelines [2].

Digital health technologies (DHTs) have introduced innovative approaches to disease management, health risk mitigation, and wellness promotion. This transformation involves wearable devices, Health Information Technology, Electronic Health Records (EHRs), telemedicine, and personalized treatments [3]. The development of Smart Healthcare Systems (SHS) has further advanced this field, integrating the Internet of Things (IoT), AI, cloud computing, big data analytics, and sensors to create a cohesive ecosystem benefiting all healthcare stakeholders [4].

The combination of IoT and AI has significantly improved congestion control, resource allocation, and decision-making in healthcare systems [5].

The COVID-19 pandemic accelerated the deployment of these SHS, which were crucial for contact tracing, remote patient monitoring, and treatment, reducing hospital overcrowding. Machine learning (ML) algorithms helped us understand viral propagation patterns, improve diagnostics, and optimize therapeutics. They also provided valuable insights for epidemic prediction models [6].

Infodemiology played a key role in managing the information overload around the pandemic, particularly regarding vaccination [7]. Social media data enabled researchers to identify and characterize post-COVID-19 condition symptoms, improving understanding of this complex syndrome [8, 9].

For mild COVID-19 cases, remote monitoring was facilitated through wearable devices, smartphone applications, internet-based drug delivery systems, and telemedicine platforms. DHTs coupled with AI enable precise prognostic predictions and provide clinicians with reliable decision support for PCC management [10]. However, comprehensive documentation on the application of these modern digital approaches remains limited.

The introduction of the U09.9 code by the Centers for Disease Control and Prevention (CDC) in October 2021 marked a significant step in documenting PCC [11]. However, this approach has limitations, including potential underestimation of prevalence, issues of residual confounding, missing data, and mis-recording [12–16].

The continuous need to identify PCC cohorts for clinical trials and manage disease-related costs has led to several studies on retrospective PCC diagnosis and prevalence estimation using EHR. These studies typically employ a predefined symptom list and compare incidence rates between SARS-CoV-2-infected patients and non-infected controls, utilizing various analytical methods, including causal inference, regression analysis, and network analysis [17].

While AI offers advantages in analysing EHRs for PCC diagnosis, ethical concerns surrounding privacy, data protection, and patient identification risks necessitate robust security measures [18]. Therefore, it is important to understand the role these innovative technologies play in ethical PCC diagnosis and management.

This review aims to critically assess the use of DHTs, AI, and infodemiology in diagnosing, predicting, estimating prevalence, monitoring, and treating PCC while addressing the associated ethical and practical challenges. By synthesizing current research and identifying knowledge gaps, we seek to provide a comprehensive understanding of the evolving landscape of digital health approaches to post-COVID care. This review will contribute to the growing literature on PCC diagnosis and management, inform future research directions, and potentially guide policy decisions in digital health interventions in chronic post-infectious conditions.

## Methods

This systematic review was conducted according to the Preferred Reporting Items for Systematic Reviews and Meta-Analyses (PRISMA) recommendations [19].

### Search strategy

A systematic literature search of articles published between January 2020 and May 2024 was performed within Scopus and PubMed. The search strategy was adapted to each of the databases as necessary. The relevant search terms were identified using medical subject heading (MeSH) phrases, and synonyms correlated to the review topic. Then, two reviewers (AM and PM) systematically searched the above-mentioned databases using the search strings developed by combining the identified search terms and Boolean operators. Only articles available in full text were included (see all inclusion criteria below).

After obtaining the articles from the database searches, two reviewers (AM and PM) removed all duplicates and independently screened the study titles and abstracts. Finally, a comprehensive evaluation of the full texts was independently performed by the two reviewers (AM and PM) to verify that the selected articles met the inclusion and exclusion criteria.

The titles and abstracts were screened independently by following inclusion and exclusion criteria. After this, the two reviewers (AM and PM) independently evaluated the full texts of the selected studies. The resulting studies were then retained for analysis.

We included peer-reviewed journal articles in PubMed and Scopus published from 1 January 2020 to 21 May 2024 in French or English, including experimental, observational, qualitative, or mixed-methods studies focusing on individuals diagnosed with PCC according to the CDC or WHO’s definition of PCC. Only studies investigating the use of DHTs, AI, or ML, and infodemiology in the management of PCC were included.

### Data extraction

**Figure 1:**
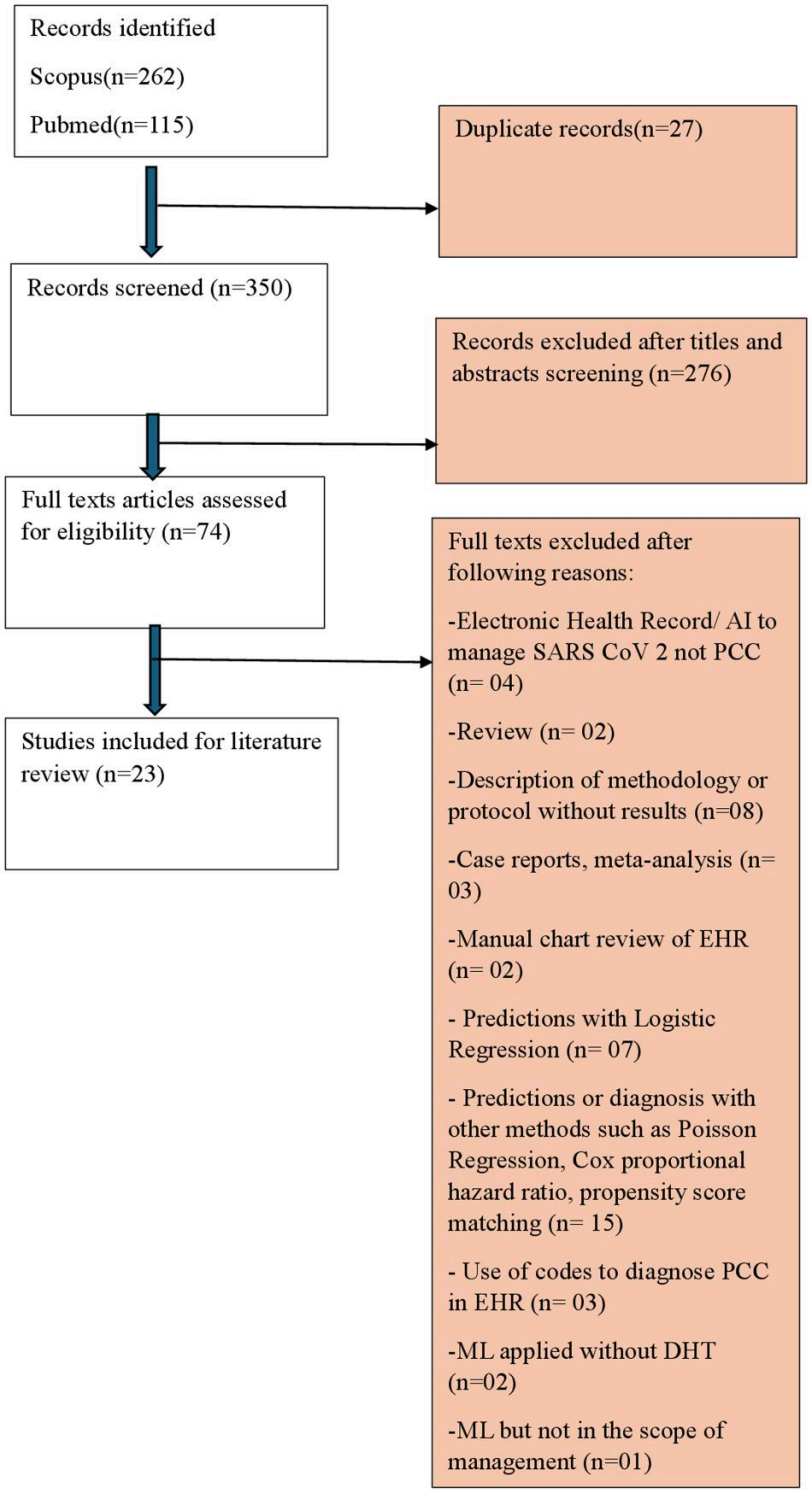
Study selection flowchart

The following data from each study were extracted to Microsoft Excel: (i) general information (authors, country), (ii) study characteristics (year, country, study design, sample size, mean or median age, percentage of female participants), (iii) assessment of symptoms; (iv) follow-up duration, and (v) outcomes. This process was carried out by two of the researchers (AM and PM). Any uncertainties were resolved through consensus with another author (YC) in case of discrepancies between reviewers during the screening and data extraction. Figure 1 illustrates the PRISMA flowchart of the study selection process and depicts the total number of retrieved, included, and excluded studies.

## Results

### Study selection

After employing the search strategy, 377 studies were found. Upon reviewing the titles and abstracts, 74 studies were included for selection. Subsequently, a review of the full text articles was conducted for a more in-depth evaluation. In the end, 23 studies were included.

### Study characteristics

Out of 23 included studies, a third were conducted in the USA (eight studies), Canada, Australia, France, Italy, Czech Republic, Scotland, Ukraine (one study each), UK (2), Poland (2), Spain (3), and one multicentred (Europe and America). They were principally observational retrospective cohort studies. The studies had differing mean or median ages, sample sizes, proportions of female participants, symptom assessments, and follow-up durations. The included studies had sample sizes varying from 12 to 84 981 participants. The mean or median age ranged between 45.93 and 65.6 years. The percentage of female participants was 22.7%–86%.

#### PCC diagnosis and prevalence estimation

PCC diagnosis was mainly conducted retrospectively, using EHR in six studies where ML was leveraged using ML for modelling Health Outcome (MLHO), often associated with chart reviews [16, 20–21], Free Text Matching Algorithm (FMA) [22], Natural Language Processing (NLP) [23], and XGBoost [24].

Deguilhem et al. used infodemiology, which leveraged a supervised ML algorithm to identify long COVID patients on social media [8]. They found that approximately 49.1% of users (79/161) continued expressing symptoms after more than 3 months post-infection, and 20.5% (33/161) after 1 year.

One study used digital devices to diagnose sub-clinical arrhythmias [25], and another one found differential gene expression targeted by miRNAs in people with DLCO (Diffusing Capacity of the Lungs for Carbon Monoxide) < 80% and DLCO> = 80%; these genes were suggestive of pulmonary fibrosis post-COVID [26] (see Table 1).

**Table 1.** Post-COVID conditions diagnosis and prevalence estimation.

Title	First author	Number of participants	Population	Tools or procedures	Diagnosis	Prevalence
A Retrospective Cohort Analysis Leveraging Augmented Intelligence to Characterize Long COVID in the Electronic Health Record: A Precision Medicine Framework	Zachary H Strasser	8344	Hospitalized	MLHO + chart review		25% (CI 95%: 6–48), 11% (CI 95%: 6–15), and 13 percent (CI 95%: 8–17) of hospitalized COVID-19 patients will have dyspnoea, fatigue, and joint pain, respectively, 3 months or longer after a COVID-19 diagnosis.
MicroRNA-Centered Theranostics for Pulmoprotection in Critical COVID-19	Perez-Pons, M.	172	172 critical COVID-19 survivors	miRNA-based prediction model.	Eight transcripts (CAV2, MAP1B, VLDLR, GSPT1, ATP1B2, ADAMTS1, CDCA7, and AKAP12) exhibited differential expression among the study groups	
Identifying Profiles and Symptoms of Patients With Long COVID in France: Data Mining Infodemiology Study Based on Social Media	Amélia Déguilhem	161	As a result, of the 128 083 retrieved messages, 15 364 messages were identified as having originated from 6494 patients with long COVID or their caregivers	Supervised ML algorithm (pipeline featuring 2 XGBoost classifiers)		Approximately 49.1% of users (79/161) continued expressing symptoms after more than 3 months post-infection, and 20.5% (33/161) after 1 year.
Identifying Who Has Long COVID in the USA: A Machine Learning Approach Using N3C Data	Pfaff, Emily R	846981	(1) all patients, (2) patients who had been hospitalized with acute COVID-19, and (3) patients who were not hospitalized.	The Python package XGBoost. Each model was run against this three-site population, resulting in AUROCs of 0.92 for the all-patients model, 0.90 for the hospitalized model, and 0.85 for the non-hospitalized model	AUROCs of 0.92 for the all-patients model, 0.90 for the hospitalized model, and 0.85 for the non-hospitalized model	
Evolving Phenotypes of Non-Hospitalized Patients That Indicate Long COVID	Estiri, Hossein	22 475	Outpatients	MLHO+chart reviews.		45.71% of the outpatient cohort who tested for the infection
Characterization of Long COVID Temporal Sub-Phenotypes by Distributed Representation Learning From Electronic Health Record Data: A Cohort Study	Dagliati, Arianna	12 424	Inpatients	MLHO		15.7 (11.12—20.03) % of the hospitalized COVID-19 patients had at least one PASC problem and 5.98 (4.06–7.91) percent had multiple problems. Joint pain and dyspnoea had an average prevalence of 5.45 (4.14–6.76) and 4.53 (3.95–5.09).
Prevalence and Risk Factors for Long COVID Among Adults in Scotland Using Electronic Health Records: A National, Retrospective, Observational Cohort Study	Jeffrey, Karen	4 676 390	In- and outpatients	NLP		0.02%–1.4%, Globally, 1.7%. clinical codes 0.02%, Free text entries 0.2% and 0.3% based on text recorded in primary care records and on sick notes the operational definition indicated prevalence of 1.4%
A Descriptive Study of the Clinical Impacts on COVID-19 Survivors Using Telemonitoring (The TeleCOVID Study)	Josephine Sau Fan Chow	16	Included in the study were patients admitted to the hospital with the diagnosis of COVID-19 between June 2021 and November 2021	Two smart, wearable/portable biosensor technologies		Sixteen patients with 81 (73.6%) valid tests were included in the analysis and amongst them seven patients were detected by artificial intelligence to have cardiac arrhythmias but not clinically symptomatic.
Long COVID Symptoms and Diagnosis in Primary Care: A Cohort Study Using Structured and Unstructured Data in The Health Improvement Network Primary Care Database	Shah, Anoop D.	553		A rule-based named entity recognition and linking algorithm called the FMA		818 records of suspected or confirmed long COVID in the free text among the cohort (553 unique patients). Among confirmed COVID at least 12 weeks prior, 103 individuals (0.9%) had a free text entry for confirmed or suspected long COVID

MLHO: Machine Learning for modeling Health Outcomes; miRNA: micro-Ribonucleic Acid; CI: Confidence Interval; CAV-2: Caveolin-2; MAP1B: Microtubule-Associated Protein 1B; VLDLR: Very Low Density Lipoprotein Receptor; GSPT1: G1 to S Phase Transition 1; ATP1B2: Adenosine Triphosphate 1 Beta 2 subunit; ADAMTS1: a Disintegrin and Metalloproteinase with Thrombospondin Motif; CDCA7: Cell Division Cycle Associated 7; AKAP12: A Kinase Anchoring Protein 12; N3C: National COVID Collaborative Cohort; XGBoost: eXtreme Gradient Boosting; AUROC: Area Under the Receiver Operator Characteristic; PASC: Post-Acute Sequalae of SARS CoV-2; NLP: Natural Language Processing; FMA: Free-Text Matching Algorithm.

Prevalence estimation was quite heterogeneous using EHR and ML. Two studies focused on symptom prevalence [21, 25]. Jeffrey et al. found a national prevalence of 0.02%–1.4%, depending on which of the four measures below was used. Long COVID clinical codes indicated a prevalence of 0.02%, possibly due to under-utilization of the codes. Free text entries suggested a higher prevalence of 0.2% and 0.3%, based on text recorded in primary care records and sick notes (respectively), while the operational definition indicated a prevalence of 1.4% [24].

#### PCC prediction

One study concluded that Random Forest (RF) and logistic regressions perform the same in predicting PCC [27]. Multiple studies used these methods [28–30]. Several studies leveraged ML algorithms to predict PCC using XGBoost, RF and Super Learner, an ensemble predictive algorithm that uses cross-validation to arrive at the optimal weighted combination of base learners.

To predict PCC and find the variables associated with a higher risk of developing the condition, one study used personalized medicine, precisely miRNAs, which are predictive of alteration of DLCO in Post-COVID [26]. Bartczak et al. used telemonitoring and found that a patient who provides at-rest saturation measurements lower than 94% will not significantly improve in pulmonary function tests—FVC (Forced Vital Capacity) and DLCO—after 2–3 months post-discharge, thus suffering continuous exercise intolerance.

Myska et al. chose among logistic regression, K-nearest neighbours, decision-tree, XGBoost, RF, Support Vector Machine, Multilayer Perceptron (MLP), AdaBoost classifier, and Light Gradient Boost Machine (LGBM). The selection of the algorithms used in the experiment was based primarily on their ability to analyse smaller datasets and their potential for easy explanation, which is particularly relevant to the decision tree algorithm. The best results were achieved by the decision tree with balanced accuracy 73.69%, F1 score—71.70%, precision—73.08%, ROC-AUC—74.69%. They found that the most important features that can predict whether a patient will suffer from pulmonary fibrosis within 3 months are the amount of corticosteroid therapy received during COVID-19 treatment and IgM values from blood tests [31].

Three studies used ML:

Antony et al. used RF and found that features such as age, gender, symptoms such as cough and fatigue during the acute COVID-19 infection, comorbidities such as chronic lung disease, depression, diabetes, kidney disease, and obesity are predictive of PCC with Area Under the Receiver Operator Characteristic (AUROC) of 0.77 (IQR = 0.01) [29].

Pfaff et al. used XGBoost and found that important predictive features included patient age, dyspnea, fatigue, and other diagnosis and medication information available within the EHR, with AUROC = 0.72 [32].

Casal-Guisande et al. used a Classification and Regression Tree (CART)-type decision tree for initial feature selection, identifying sex, age, lung diseases, smoking, presence of dyspnoea in the third month, and diffusing capacity of the lungs for nitric oxide as key variables. However, their approach goes beyond ML, employing a cascade of expert systems with knowledge bases constructed using the Wang-Mendel automatic rule generation algorithm. Importantly, these knowledge bases were subsequently reviewed and validated by medical professionals. This hybrid approach combines ML for feature selection with symbolic AI methods, specifically focusing on predicting dyspnoea at 12 months in relation to long COVID—a targeted outcome highly relevant for pulmonologists. Their system achieved an Area Under the Curve (AUC) of 0.75 [33].

A Superlearner model incorporated five types of learners, including RF, Generalized Linear Model, elastic net, XGBoost, and Bagging Classification Trees and found that the models utilizing pre-COVID clinical data period, including variables like hypertension, were identified as diagnoses predictive of PCC. During the acute COVID period, predictive signs and symptoms mainly revolved around the respiratory system, including shortness of breath and respiratory abnormalities. Additionally, symptoms such as malaise, fatigue and chronic fatigue syndrome significantly contributed to the prediction of PCC Additionally, malaise, fatigue, and chronic fatigue syndrome contributed to PCC prediction and diagnoses in the acute COVID-19 period have a stronger and more stable contribution to prediction. [34] (Table 2).

**Table 2.** Post-COVID conditions prediction.

Title	First author	Number of participants	Population	Tools or procedures	Results/Performance
MicroRNA-Centered Theragnostic for Pulmoprotection in Critical COVID-19	Perez-Pons, M.	172	172 critical COVID-19 survivors	miRNA-based prediction model	The optimal model included a 3-miRNA signature composed of miR-27a-3p, miR-93-5p, and miR-199a-5p
Predictive Models of Long COVID	Antony, Blessy	17 036	In- and outpatients	SHAP (SHapely Additive exPlanations)+ LR and RF	LR and RF had virtually the same performance in all three cohorts with median AUROC and IQR between 0.74 (IQR=0.01) and 0.77 (IQR=0.01), and median AUPRC and IQR between 0.02 (IQR=0.00) and 0.08 (IQR=0.01)
Using Multi-Modal Electronic Health Record Data for the Development and Validation of Risk Prediction Models for Long COVID Using the Super Learner Algorithm	Jin, Weijia	2287 received a diagnosis of PASC.		Super Learner (SL)-based predictor. The SL model incorporated five types of learners, including random forest, Generalized Linear Model, elastic net, XGBoost, and Bagging Classification Trees.	AllRS moderately predicted PASC (AAUC(AllRS): 0.64 (0.6, 0.68)), its discrimination power did not demonstrate a substantial improvement compared to the individual risk scores (AAUC(PheRS): 0.64 (0.59, 0.68))
Artificial-Intelligence-Driven Algorithms for Predicting Response to Corticosteroid Treatment in Patients with Post-Acute COVID-19	Myska, Vojtech	281 The patients are split into two groups: (1) patients who received CS therapy as post-COVID treatment (95) and (2) those who did not (186)	In patients and outpatients	logistic regression, K-nearest neighbours, decision-tree, XGBoost, random forest, Support Vector Machine, MLP, AdaBoost classifier, LGBM	According to the results presented the best results are achieved by the decision tree with balanced accuracy 73.69%, F1 score—71.70%, precision—73.08%, ROC-AUC—74.69%.
The Utility of Telemedicine in Managing Patients After COVID-19	Bartczak, Krystian T	30	Inpatients	telemedicine equipment consisting of pulse oximeters (MIR Spirotel^®^) connected with Samsung Galaxy Tab 3 tablets	a patient who provides at-rest saturation measurements lower than 94% will not significantly improve in pulmonary function tests—FVC and DLCO—after 2 to 3 months post-discharge. This equals continuous exercise intolerance.
De-Black-Boxing Health AI: Demonstrating Reproducible Machine Learning Computable Phenotypes Using the N3C-RECOVER Long COVID Model in the All of Us Data Repository	Emily R Pfaff	8998		Python package XGBoost	AUROCs (N3C= 0.83 and All of Us = 0.72)
Proposal and Definition of an Intelligent Clinical Decision Support System Applied to the Prediction of Dyspnea after 12 Months of an Acute Episode of COVID-19	Casal-Guisande, Manuel	185	Inpatients	a CART-type decision tree	AUC = 0.75

miRNA: micro-Ribonucleic Acid; SHAP: SHapely Additive Explanation; LR: Logistic Regression; RF: Random Forest; AUROC: Area Under the Receiver Operating Characteristic; IQR: Interquartile Range; AUPRC: Area Under the Precision-Recall Curve; PASC: Post-Acute Sequalae of SARS CoV-2; SL: Super Learner; XGBoost: eXtreme Gradient Boosting; AAUC: Area under the Covariate-Adjusted Receiver Operating Characteristic; PheRS: Phenotype Risk Score; AllRS: All Risk Score; CS: Corticosteroid Therapy; MLP: Multilayer Perceptron; LGBM: Light Gradient Boost Machine; ROC: Receiver Operating Characteristic; AUC: Area Under the ROC Curve; N3C: National COVID Cohort Collaborative; CART-type Decision Tree: Classification and Regression Tree.

#### PCC monitoring

To date, PCC duration is not well defined. Deguilhem et al. monitored the symptom duration of some patients on social media, and 20.5% still had symptoms one year after the infection [8]. Monitoring also focused essentially on specific symptoms of conditions associated with PCC, such as signs and symptoms of cardiovascular diseases (CVD) [35] and of the pulmonary disorders (cough and dyspnoea) [36]. Sivan et al. devised a digital patient-reported outcome measure (DPROM) platform to monitor functional physical and functional outcomes of the condition and patient-reported outcomes measure (PROM) and patient-reported experience measures (PREM) to assess symptoms’ severity and fluctuation [37]. More sophisticated and innovative are embedded sensors which monitor patients’ performances and progress remotely by counting the number of exercise repetitions correctly performed, which is an output of the developed AI algorithm [38] (Table 3).

**Table 3.** Post-COVID conditions monitoring.

Title	First author	Number of participants	Population	Tools or procedures	Patterns monitored
Identifying Profiles and Symptoms of Patients With Long COVID in France: Data Mining Infodemiology Study Based on Social Media	Amélia Déguilhem	161	As a result, of the 128 083 retrieved messages, 15 364 messages were identified as having originated from 6494 patients with long COVID or their caregivers	Supervised ML algorithm (pipeline featuring 2 XGBoost classifiers)	PCC symptoms and evolution
A Descriptive Study of the Clinical Impacts on COVID-19 Survivors Using Telemonitoring (The TeleCOVID Study)	Josephine Sau Fan Chow	16	Included in the study were patients admitted to the hospital with the diagnosis of COVID-19 between June 2021 and November 2021	two smart, wearable/portable biosensor technologies	SpO_2_, ECG, symptoms of CVD
The Utility of Telemedicine in Managing Patients After COVID-19	Bartczak, Krystian T	30	Inpatients	telemedicine equipment consisting of pulse oximeters (MIR Spirotel^®^) connected with Samsung Galaxy Tab 3 tablets	SpO_2_, HR, cough, dyspnoea
Virtual Pulmonary Rehabilitation Approaches in Patients With Post COVID Syndrome: A Pilot Study	Sarmento, Antonio	14 participants: 8 in the PRVC and 6 in the PRSD	In- and outpatients (93%)	PRVC or PRSD exercises.	Maximum heart rate and SpO_2_ during exercise
Digital Patient Reported Outcome Measures Platform for Post-COVID-19 Condition and Other Long-Term Conditions: User-Centered Development and Technical Description	Manoj Sivan	10 000		A DPROM platform	Symptoms fluctuation and severity
Telerehabilitation with ARC Intellicare to Cope with Motor and Respiratory Disabilities: Results about the Process, Usability, and Clinical Effect of the “Ricominciare” Pilot Study	Marianna Capecci	Of the 23 subjects receiving training sessions (12 COV19 and 11 pwPD)		ARC, a telerehabilitation solution based on the use of multiple wearable sensors, a mobile device, and algorithms of artificial intelligence (patent pending).	Exercise monitoring with sensors

XGBoost: eXtreme Gradient Boosting; PCC: Post-COVID Conditions; SpO2: Oxygen Saturation; ECG: Electrocardiogram; CVD: Cardiovascular Disease; HR: Heart Rate; PR: Pulmonary Rehabilitation; PRVC: Pulmonary Rehabilitation Through Videoconference; PRSD: Pulmonary Rehabilitation Self-Directed; DPROM: Digital Patient-Reported Outcome Measure; COV19: People With COVID-19; pwPD: People With Parkinson Disease; ARC: An Artificial Intelligence-Powered and Inertial Motion Unit-Based Mobile Platform.

#### PCC management

Out of 10 studies on the management of PCC, eight focused on personalized medicine, including:

A study used theragnostic agents, miRNAs [26], while two others used AI-based patient-centred decision-making [31, 33]. In addition, one leveraged AI-assisted telerehabilitation assessment [38], and another focused on neurological symptoms with cognitive behavioural therapy (CBT) [39]. Several studies focused on respiratory, cardiovascular, and motor symptoms (2) [40, 41], physical and functional disabilities (1) [39], and physiotherapy delivered remotely to patients (2) [42, 43] (see Table 4).

**Table 4.** Post-COVID conditions management.

Title	First author	Number of participants	Population	Tools or procedures	Patterns assessed
MicroRNA-Centered Theranostics for Pulmoprotection in Critical COVID-19	Perez-Pons, M.	172	172 critical COVID-19 survivors	miRNA-based prediction model	Among the 7072 target genes, 1133 were proved to be targets of at least two miRNAs
Artificial-Intelligence-Driven Algorithms for Predicting Response to Corticosteroid Treatment in Patients with Post-Acute COVID-19	Myska, Vojtech	281 The patients are split into two groups, (1) patients who received CS therapy as post-COVID treatment (95) and (2) those who did not (186)	In patients and outpatients	logistic regression, K-nearest neighbours, decision-tree, XGBoost, random forest, Support Vector Machine, MLP, AdaBoost classifier, LGBM	artificial intelligence-based algorithms for personalizing CS treatment in patients with a risk of developing pulmonary fibrosis due to COVID-19.
Virtual Pulmonary Rehabilitation Approaches in Patients With Post COVID Syndrome: A Pilot Study	Sarmento, Antonio	14	In- and outpatients (93%)	Eight-week virtual PR programme in which the same exercise components were delivered via group sessions (PRVC) or self-directed (PRSD).	Feasibility, safety, adherence, satisfaction, effectiveness of PR on lung function, dyspnoea, fatigue, sit-to-stand capacity, HRQoL, and participation of individuals with PCS-related respiratory symptoms.
Digital Patient Reported Outcome Measures Platform for Post-COVID-19 Condition and Other Long-Term Conditions: User-Centered Development and Technical Description	Manoj Sivan	10 000		A DPROM platform	A platform to record PCC symptom profile, condition severity, functional disability, and quality of life via the C19-YRS and other PROMs within the platform. The platform generates easy-to-understand scores, radar plots, and line graphs for people with PCC to self-monitor their condition and assess response to interventions.
Gender-Related Effectiveness of Personalized Post-COVID-19 Rehabilitation	Rzepka-Cholasińska, Alicja	90	In- and outpatients	Telerehabilitation	A high-intensity 6-week exercise performed three times per week, except resistance exercises, which were performed once a week in the initial two weeks, twice a week in the following two weeks, and three times in the final two weeks of the rehabilitation programme.
Pilot Observational Study of Patient Reported Outcome Measures for Long COVID Patients in Virtual Integrative Medical Group Visits	Barnhill, Jessica L	14		IMGVs (Integrative Medical Group visits) via Telehealth.	Experiential activities include mindfulness mediations (awareness of breath meditation, body scan, mindful eating, sitting meditation, chair yoga, and loving kindness meditation), self-acupressure, gratitude journaling, self-massage, and goal setting, as well as anti-inflammatory diet recipes associated to health education with a focus on neuroplasticity and energy pacing.
The Effectiveness of a Four-Week Digital Physiotherapy Intervention to Improve Functional Capacity and Adherence to Intervention in Patients with Long COVID-19	Estebanez-Pérez, María-José	32	In- and outpatients (90.6%)	A 4-week personalized digital physiotherapy programme of 1 session per day of 45–50 min maximum and always adapted to the previous evaluation and to the needs of each patient.	Personalized recommendations for each patient like walking, jogging or swimming added to the supervised digital interventions based on individual patient needs, progressive strength training and secretion drainage or ventilatory techniques
Proposal and Definition of an Intelligent Clinical Decision Support System Applied to the Prediction of Dyspnea after 12 Months of an Acute Episode of COVID-19	Casal-Guisande, Manuel	185	Inpatients	A CART-type decision tree	Personalized follow-up process with individualized specific studies is established for possible cases of dyspnoea at 12 months.
Provision of Medical-psychological and Psychiatric Care to Patients with Post-covid Syndrome in Telemedicine Conditions	Koliadenko, Nina V	129		Telerehabilitation	CBT and psychology and psychopharmacological treatment
Telerehabilitation with ARC Intellicare to Cope with Motor and Respiratory Disabilities: Results about the Process, Usability, and Clinical Effect of the “Ricominciare” Pilot Study	Marianna Capecci	Of the 23 subjects receiving training sessions (12 COV19 and 11 pwPD)		ARC, a telerehabilitation solution based on the use of multiple wearable sensors, a mobile device, and algorithms of artificial intelligence (patent pending).	Independently at home for 4 weeks, for 45 min 5 days/week sessions of respiratory and motor patient-tailored rehabilitation.

MiRNA: micro-Ribonucleic Acid; XGBoost: eXtreme Gradient Boosting; MLP: Multilayer Perceptron; LGBM: Light Gradient Boost Machine; CS: Corticosteroid Therapy; PR: Pulmonary Rehabilitation; PRVC: Pulmonary Rehabilitation through Video Conference; PRSD: Pulmonary Rehabilitation Self-Directed; HRQoL: Health-Related Quality of Life; PCS: Post-COVID Syndrome; PCC: Post-COVID Conditions; C19-YRS: COVID-19 Yorkshire Rehabilitation Scale; DPROM: Digital Patient-Reported Outcome Measures Platform; PROMs: Patient Reported-Outcome Measures; IMGVs: Integrative Medical Group Visits; CART: Classification and Regression Tree; CBT: Cognitive Behavioural Therapy; ARC: an artificial intelligence-powered and inertial motion unit-based mobile platform.

## Discussion

This review aimed to critically assess the use of DHTs, AI, and infodemiology in diagnosing, predicting, estimating prevalence, monitoring, and treating PCC while addressing the associated ethical and practical challenges. Studies that described modern digital health approaches used to manage PCC involved EHRs coupled with ML algorithms, mobile devices coupled with AI or not and often embedded with sensors for teleassessment/telemonitoring and telerehabilitation, theragnostic agents, AI-based personalized medicine or simple personalized medicine, and infodemiology. Almost a third of those studies originated from North America, particularly the USA and over half from Europe, with none from low- and middle-income countries (LMICs).

### Benefits and limitations of DHTs

DHTs can facilitate data-driven decision-making in clinical settings. Integrating AI and ML algorithms into digital health applications enables healthcare providers to analyse large datasets effectively, improving diagnostic and therapeutic strategies [44]. For instance, ML techniques have been employed to identify immune signatures associated with PCC-like symptoms, revealing potential biomarkers that could predict the severity and chronicity of symptoms [45]. In addition, digital platforms can aggregate data from diverse sources, enhancing predictive modelling and helping healthcare providers anticipate complications and tailor treatment plans accordingly [46]. For example, wearable devices can collect real-time physiological data, which can be invaluable for tracking symptoms and disease progression in PCC patients [47].

Furthermore, AI can assist in aggregating and analysing patient-reported outcomes, which are critical in understanding the subjective experience of PCC symptoms. By employing NLP techniques, AI can analyse free-text entries from patient EHRs regarding their symptoms, providing insights into the frequency, duration, and severity of various manifestations of PCC[48].

AI algorithms can enhance the monitoring of patients through wearable devices that continuously collect health data, such as heart rate, oxygen saturation, and physical activity levels. These devices can provide real-time feedback to both patients and healthcare providers, facilitating timely adjustments to treatment plans based on the patient's status [46]. Additionally, remote monitoring reduces the need for in-person visits, minimizing exposure risks for patients and healthcare providers [49].

DHTs also offer innovative treatment modalities, including teletherapy, virtual rehabilitation programmes, and digital therapeutics. Telehealth platforms allow patients to access mental health support and physical rehabilitation services from the comfort of their homes, which is particularly beneficial for those experiencing mobility issues or fatigue [50]. Digital therapeutics, such as CBT apps, can provide evidence-based interventions tailored to individual needs, enhancing treatment adherence and outcomes [51]. Furthermore, using vocal biomarkers to predict fatigue and other symptoms in people with COVID-19 is a novel approach to assessing patient progress and adjusting treatment strategies [52].

Finally, implementing DHTs can lead to cost savings for healthcare systems by reducing the need for hospital visits and enabling more efficient resource allocation [53]. These technologies enhance accessibility to care, particularly for underserved populations who may face barriers to traditional healthcare services [54]. By providing remote access to healthcare professionals, DHTs may ensure that patients with conditions like PCC receive timely support, regardless of their geographical location, as has been shown with neglected tropical diseases and other non-communicable diseases [55].

Nonetheless, these technologies have limitations. One of the primary limitations of DHTs is the disparity in digital literacy among patients. Many individuals, particularly older adults and those from lower socioeconomic backgrounds, may lack the necessary skills to effectively use digital health tools [55, 56]. This digital divide can exacerbate health inequities, as those with limited access to technology or lower digital literacy may not benefit from digital health interventions [57]. Furthermore, issues such as limited internet connectivity and smartphone access can further restrict the reach of digital health solutions, particularly in rural or underserved areas [56, 57]. This divide can lead to underrepresentation of certain demographics in digital health initiatives, limiting the generalizability of findings and interventions [58].

Another challenge is the integration of DHTs with existing healthcare systems. Many healthcare providers may lack the necessary training and resources to effectively implement and utilize these technologies in their practice [55, 56]. This gap can lead to inconsistencies in care delivery and hinder the potential benefits of digital health solutions [57, 59]. In addition, models trained on EHRs data should consider the underrepresentation of certain patient groups, such as those who are uninsured, have limited access to care, or seek treatment at small practices or community hospitals with limited data exchange capabilities [24].

The efficacy of DHTs can vary significantly based on the specific tools and applications used. While some technologies may demonstrate positive outcomes in managing PCC symptoms, others may lack robust evidence supporting their effectiveness [60]. This variability can lead to confusion among healthcare providers and patients regarding which digital interventions are most beneficial, potentially resulting in suboptimal treatment choices [60, 61].

DHTs may not adequately address the psychological and social aspects of PCC. Many patients report feelings of isolation and lack of support, which can be exacerbated by reliance on digital interactions rather than in-person care [60].

Moreover, reliance on DHTs raises data privacy and security concerns. The collection and storage of sensitive health information through digital platforms can expose patients to risks of data breaches and unauthorized access, which may deter individuals from using these technologies [62]. Additionally, the quality of information provided through digital health platforms can vary significantly, leading to potential misinformation and mismanagement of health conditions [62]. The security and ethical concerns related to the use of DHTs are further discussed below.

In conclusion, while DHTs offer promising avenues for managing PCC, their limitations must be addressed to maximize their effectiveness and ensure equitable access for all patient groups.

### Digital approaches in PCC management

Based on this review, we grouped the digital approaches to managing PCC into four groups: AI and EHR associations, AI-based patient-centred decision-making, infodemiology, and telemonitoring/telerehabilitation with digital devices.

#### AI and EHR associations

AI automates data extraction and analysis, thus significantly reducing the time required for manual review and effectively managing large amounts of data. ML models have been trained on large datasets from EHR to identify key risk factors for PCC symptoms, such as respiratory problems, fatigue, and pre-existing conditions. By analysing patterns in patient data, AI models provide valuable insights that help healthcare providers prioritize high-risk patients and tailor interventions more effectively.

Although these technologies are not extensively trained for PCC, they have been successfully implemented in other areas, such as cancer, improving accuracy by minimizing human errors and identifying complex patterns. This ensures consistency and standardization in documentation across different clinicians and health facilities [63].

To identify patterns of PCC within EHRs using ML, it is necessary to utilize methods like NLP [24], FMA, a rule-based method [22], and in the future, Large Language Models (LLM). Although FMA is efficient for finding specific data points in EHRs, it may struggle with variations in language or complex medical terminology. Therefore, Free-text analysis is always subject to error because no computer algorithm can always interpret the nuances of human language correctly. Thus, there may have been false negatives and false positives in reporting symptoms, with a potential risk of bias due to misclassification [64]. NLP is more advanced and can handle variations in language and identify relevant information even if it is not expressed using exact keywords. Moreover, NLP can automate de-identification of protected health information [65]. LLM, an advanced NLP, are well-suited for discerning subtle indicators of PCC in EHR text because they can understand complex relationships and nuances in language.

The development and validation of ML algorithms requires extensive datasets with clearly defined PCC cases to ensure accuracy and generalizability. A study by Pfaff et al. elucidated the challenges of translating a model from one dataset to another, including peculiarities in coding, the absence of low-prevalence concepts, and missing features contributing to the results' differences [32].

MLHO offer the potential to detect rare associations and understand complex non-linear relationships [21]. MLHO aimed to verify if clinical notes matched ICD-9/10 labels when associated with chart review. Physicians frequently linked alopecia and anosmia/ageusia to COVID-19 but did not make this connection for other phenotypes like diabetes or chest pain. Their model found that many unrecognized phenotypes still had high confidence scores. While ICD codes do not indicate onset time, the chart review confirmed that these phenotypes likely appeared post-COVID [20]. The MLHO framework outperforms univariate PheWAS (Phenome-wide association studies) using a comprehensive, multivariate approach, reducing false positives. MLHO's algorithms evaluated over 1600 phenotypes, identifying those associated with COVID-19. This study also debunked some previously identified phenotypes, like alopecia and cutaneous eruption outside of the nails, by including COVID-negative patients for comparison [20].

According to Pfaff et al., who used the XGBoost ML algorithm, EHRs offered the advantage of understanding the complexities of PCC by recruiting a large and diverse cohort of research participants, which was necessary. Efficient recruitment of cohorts of this size often involves using computable phenotypes (electronic cohort definitions) to identify patients who meet the study's inclusion criteria, as poor cohort definitions can lead to poor study outcomes [24]. For PCC, the lack of a clear consensus definition and the condition's heterogeneity pose a significant challenge to cohort identification. ML can help address this challenge by using the rich longitudinal data available in EHRs to identify patients similar to those with PCC algorithmically [24]. EHR data are well-suited for cohort definition through computable phenotyping, especially for study recruitment. While there are other methods of identifying potential study participants, a computable phenotype allows for efficiently narrowing down the recruitment pool to patients who are likely to qualify. This eliminates many patients who do not qualify (false positives) and identifies patients who may not be identified through human curation (false negatives) [24]. With an evolving definition and no gold standard for comparison, the EHR allows for defining proxies for the condition. Instead of relying solely on a restrictive criterion of at least one visit to a long COVID speciality clinic, ML models can decouple patients' utilization patterns from clinic visits. This means the models can identify similar patients who may not have access to a long COVID clinic [24].

Similarly, Super Learner, another ML algorithm, demonstrated superior performance compared to individual ML algorithms when associated with EHRs in predicting PCC [34].

#### AI-based patient-centred decision-making

Symbolic AI approaches, particularly expert systems, in predicting PCC outcomes offer significant advantages over black-box ML methods [33, 66]. Unlike opaque neural networks or complex ensemble models, symbolic approaches provide full interpretability, allowing for clear explanations of the system's decision-making process [67]. This transparency is crucial in medical applications, where understanding the reasoning behind predictions is essential for clinical trust and adoption. With their rule-based logic, expert systems enable clinicians to trace and verify each step of the inference process, facilitating easier validation by domain experts [67]. The integration of automatic rule generation techniques, such as the Wang-Mendel algorithm, further enhances the utility of symbolic approaches by enabling the creation of knowledge bases from numerical data while maintaining interpretability [33].

While black-box models may achieve high accuracy in some cases, they cannot often provide clear explanations for their predictions, which can be problematic in healthcare settings [68, 69]. In contrast, implementing interpretable DHTs in post-COVID care based on symbolic AI shows great promise for enhancing patient monitoring and management. These approaches can be integrated with various data sources, including AI-powered symptom tracking apps, telemedicine platforms with wearable devices, and EHRs, to create comprehensive and explainable models for monitoring, predicting, and managing long-term COVID-19 sequelae [67, 69]. The optimal approach may involve a hybrid model that combines the strengths of both methodologies, leveraging the interpretability of symbolic methods alongside the predictive capabilities of black-box models [70]. These symbolic AI-driven digital approaches offer advantages such as continuous data collection, reduced burden on healthcare systems, improved access to care for diverse populations, and the ability to provide clear, understandable reasoning for their outputs. However, it is essential to balance these benefits with ethical considerations, particularly regarding data privacy and security, which can be more readily addressed with the transparent nature of symbolic AI systems.

#### Infodemiology

The analysis of internet data, known as infodemiology, has provided a unique lens for understanding the prevalence and characteristics of PCC. By monitoring online discussions, researchers have identified common symptoms and tracked their duration. This method captures real-time insights from a large population, offering a faster and more comprehensive understanding of PCC [71].

Infodemiology was used to diagnose, estimate the prevalence, and monitor PCC [8]. This study employed a comprehensive approach combining various data analysis techniques from multiple social media sources (e.g., Twitter, Reddit, Doctissimo, Facebook, and other forums) and finding a PCC prevalence similar to those in meta-analysis [8]. This shows that social listening can inform large-scale studies with rapidly flowing data, offering real-time patient insights [72].

#### Telemonitoring/telerehabilitation with digital devices

Telemedicine and telerehabilitation programmes allow patients to receive care and perform exercises from home, while AI-powered systems monitor their progress and adjust their treatment plans as needed [73]. These remote care options are especially valuable for patients with respiratory and motor impairments, ensuring they receive ongoing support without needing to visit healthcare facilities in person.

Using digital devices equipped with sensors allows for remote tracking of behavioural and physiological parameters such as temperature, pulse, and oxygen saturation, enabling patients and healthcare workers to monitor patients' conditions and intervene when necessary [74].

Monoj et al. conducted a study on developing a DPROM platform. This platform records patient-reported symptoms, the severity of their condition, functional disability, and quality of life using the Yorkshire Rehabilitation Scale (C19-YRS), including other PROMs and PREMs. The platform generates scores, radar plots, and line graphs for PCC patients and healthcare professionals to monitor their condition [37]. The summary report from the platform can be uploaded to the EHRs of PCC patients [37].

A study by et Chow et al. in Australia combined two smart, wearable biosensor technologies to remotely identify clinical signs and symptoms of CVD in patients previously admitted to the Intensive Care Unit for COVID-19. The device detected changes in cardiac rhythm and oxygen saturation [25]. Another study found that wearable devices could predict continuous exercise intolerance by identifying patients with resting oxygen saturation measurements lower than 94%, indicating poor pulmonary function test outcomes 2–3 months after discharge. These wearable devices provide additional benefits, such as minimizing exposure to contagious patients and enabling remote training for multidisciplinary health teams. Furthermore, telemedicine-enhanced home care improved patients' psychological well-being and facilitated tailored treatment decisions [38].

### Ethical issues, safety, and security concerns

While no studies raised concerns about safety and security during their different processes, one systematic review reported distrust of infodemics and digital tools (including for contact tracing) during the COVID-19 pandemic, primarily due to storing data on central servers, which have security issues [75]. A significant issue is the sensitivity of health data, which raise privacy concerns when digitized [76]. Governments struggle with managing and protecting this data effectively [77]. A study revealed that among DHTs, EHRs, wireless infusion pumps, endoscope cameras, and radiology information systems were the most vulnerable to attacks [78].

A few studies highlighted ethical issues, including de-identifying free text and pulling information from the free text from EHRs because the text might contain personal details that can identify someone. This can be mitigated by using an automated system to remove direct identifiers (like names and birthdates) from the text before using the FMA tool, which has been proven to work well with primary care text. However, even then, there might be 2% of personal information which is not protected [22].

Finally, the ethical issue of consent is prevalent as many users may not fully understand the terms and conditions when they agree to them [79]. Currently, no standard guidelines exist for discussing cybersecurity risks during informed consent, leaving patients potentially unaware of these dangers [80]. Although researchers may be granted permission to access, within a secure, trusted research environment, whole-population, de-identified data from EHRs for surveillance, patients may not be aware of the risks associated with the de-identification processes used [24].

### Study limitations

The limitations of this review stem primarily from the variability in study outcomes and methodologies. There was a clear geographical bias, with most studies originating from North America and Europe, with none from LMICs, limiting the global applicability of the findings. Additionally, ethical considerations and safety and security concerns were not sufficiently reported in the studies reviewed.

## Conclusion

Multiple DHTs are available for PCC management and offer significant potential for enhancing healthcare delivery and research. Future research should focus on rigorous comparative studies, including in LMICs, while addressing these technologies' ethical and security concerns. It is crucial to leverage the benefits of DHTs while ensuring equitable access and maintaining patient privacy and data security.

## Data Availability

The data presented in this study are available on request from the corresponding author

## References

[1] WHO. Coronavirus disease (COVID-19): *Post-COVID-19 Condition.* https://www.who.int/news-room/questions-and-answers/item/coronavirus-disease-(covid-19)-post-covid-19-condition: World Health Organization (3 July 2024, date last accessed).

[2] Senbekov M , Saliev T, Bukeyeva Z et al The recent progress and applications of digital technologies in healthcare: a review. Int J Telemed Appl 2020;2020:8830200. 33343657 10.1155/2020/8830200 PMC7732404

[3] Ronquillo Y , Meyers A, Korvek SJ. Digital Health. StatPearls. Treasure Island (FL) Ineligible Companies. Disclosure: Arlen Meyers Declares no Relevant Financial Relationships with Ineligible Companies. Disclosure: Scott Korvek Declares no Relevant Financial Relationships with Ineligible Companies.: StatPearls Publishing Copyright © 2024, StatPearls Publishing LLC.; 2024.

[4] Saba Raoof S , Durai MAS. A comprehensive review on smart health care: applications, paradigms, and challenges with case studies. Contrast Media Mol Imaging 2022;2022:4822235. 36247859 10.1155/2022/4822235 PMC9536991

[5] Kallam S , Chakrabarti P, Hung BT, Siva Shankar S. Remote health monitoring IoT framework using machine learning prediction and advanced artificial intelligence (AI) model. Ijritcc 2023;11:42–7.

[6] Tiwari S , Chanak P, Singh SK. A review of the machine learning algorithms for Covid-19 case analysis. IEEE Trans Artif Intell 2023;4:44–59. 36908643 10.1109/TAI.2022.3142241 PMC9983698

[7] Adebesin F , Smuts H, Mawela T et al The role of social media in health misinformation and disinformation during the COVID-19 pandemic: bibliometric analysis. JMIR Infodemiology 2023;3:e48620. 37728981 10.2196/48620 PMC10551800

[8] Déguilhem A , Malaab J, Talmatkadi M et al Identifying profiles and symptoms of patients with long COVID in France: data Mining infodemiology study based on social media. JMIR Infodemiology 2022;2:e39849. 36447795 10.2196/39849 PMC9685517

[9] Santarossa S , Rapp A, Sardinas S et al Understanding the #longCOVID and #longhaulers conversation on Twitter: multimethod study. JMIR Infodemiology 2022;2:e31259. 35229074 10.2196/31259 PMC8867393

[10] Yang D-M , Chang T-J, Hung K-F et al Smart healthcare: a prospective future medical approach for COVID-19. J Chin Med Assoc 2023;86:138–46. 36227021 10.1097/JCMA.0000000000000824 PMC9847685

[11] McGrath LJ , Scott AM, Surinach A et al Use of the postacute sequelae of COVID-19 diagnosis code in routine clinical practice in the US. JAMA Netw Open 2022;5:e2235089. 36201207 10.1001/jamanetworkopen.2022.35089 PMC9539719

[12] Taquet M , Harrison PJ. Exposure to phenytoin associates with a lower risk of post-COVID cognitive deficits: a cohort study. Brain Commun 2022;4:fcac206. 35999838 10.1093/braincomms/fcac206 PMC9384796

[13] Casey JA , Schwartz BS, Stewart WF et al Using electronic health records for population health research: a review of methods and applications. Annu Rev Public Health 2016;37:61–81. 26667605 10.1146/annurev-publhealth-032315-021353 PMC6724703

[14] Cowie MR , Blomster JI, Curtis LH et al Electronic health records to facilitate clinical research. Clin Res Cardiol 2017;106:1–9. 10.1007/s00392-016-1025-6 PMC5226988 27557678

[15] Jetley G , Zhang HE. Electronic health records in IS research: quality issues, essential thresholds and remedial actions. Decision Support Systems 2019;126:113137. 10.1016/j.dss.2019.113137

[16] Dagliati A , Strasser ZH, Hossein Abad ZS, Consortium for Clinical Characterization of COVID-19 by EHR (4CE) et al Characterization of long COVID temporal sub-phenotypes by distributed representation learning from electronic health record data: a cohort study. eClinicalMedicine 2023;64:102210. 37745021 10.1016/j.eclinm.2023.102210 PMC10511779

[17] Zang C , Zhang Y, Xu J et al Data-driven analysis to understand long COVID using electronic health records from the RECOVER initiative. Nat Commun 2023;14:1948. 37029117 10.1038/s41467-023-37653-z PMC10080528

[18] Ibrahim M , Al-Wadi A, Elhafiz R. Security analysis for smart healthcare systems. Sensors 2024;24:3375. 10.3390/s24113375 38894166 PMC11175093

[19] Page MJ , Moher D, Bossuyt PM et al PRISMA 2020 explanation and elaboration: updated guidance and exemplars for reporting systematic reviews. Bmj 2021;372:n160. 33781993 10.1136/bmj.n160 PMC8005925

[20] Estiri H , Strasser ZH, Brat GA, Consortium for Characterization of COVID-19 by EHR (4CE) et al Evolving phenotypes of non-hospitalized patients that indicate long COVID. BMC Med 2021;19:249. 34565368 10.1186/s12916-021-02115-0 PMC8474909

[21] Strasser ZH , Dagliati A, Shakeri Hossein Abad Z, Consortium for Clinical Characterization of COVID-19 by EHR (4CE) et al A retrospective cohort analysis leveraging augmented intelligence to characterize long COVID in the electronic health record: a precision medicine framework. PLOS Digit Health 2023;2:e0000301. 37490472 10.1371/journal.pdig.0000301 PMC10368277

[22] Shah AD , Subramanian A, Lewis J et al Long Covid symptoms and diagnosis in primary care: a cohort study using structured and unstructured data in The Health Improvement Network primary care database. PLoS One 2023;18:e0290583. 37751444 10.1371/journal.pone.0290583 PMC10521988

[23] Jeffrey K , Woolford L, Maini R et al Prevalence and risk factors for long COVID among adults in Scotland using electronic health records: a national, retrospective, observational cohort study. EClinicalMedicine 2024;71:102590. 38623399 10.1016/j.eclinm.2024.102590 PMC11016856

[24] Pfaff ER , Girvin AT, Bennett TD, N3C Consortium et al Identifying who has long COVID in the USA: a machine learning approach using N3C data. Lancet Digit Health 2022;4:e532–e41. 35589549 10.1016/S2589-7500(22)00048-6 PMC9110014

[25] Chow JSF , D’Souza A, Ford M et al A descriptive study of the clinical impacts on COVID-19 survivors using telemonitoring (The TeleCOVID Study). Front Med Technol 2023;5:1126258. 10.3389/fmedt.2023.1126258 PMC10067568 37020492

[26] Perez-Pons M , Molinero M, Benítez ID et al MicroRNA-centered theranostics for pulmoprotection in critical COVID-19. Mol Ther Nucl Acids 2024;35:10.1016/j.omtn.2024.102118 PMC10834986 38314095

[27] Antony B , Blau H, Casiraghi E, N3C consortium et al Predictive models of long COVID. EBioMedicine 2023;96:104777. 37672869 10.1016/j.ebiom.2023.104777 PMC10494314

[28] Nasir M , Cook N, Parras D et al Using data science and a health equity lens to identify long-COVID sequelae among medically underserved populations. J Health Care Poor Underserved 2023;34:521–34. 37464515 10.1353/hpu.2023.0047

[29] Kulenovic A , Lagumdzija-Kulenovic A. Using logistic regression to predict long COVID conditions in chronic patients. Stud Health Technol Inform 2022;295:265–8. 35773859 10.3233/SHTI220713

[30] Fritsche LG , Jin W, Admon AJ, Mukherjee B. Characterizing and predicting post-acute sequelae of SARS CoV-2 infection (PASC) in a large academic medical center in the US. JCM 2023;12:1328. 10.3390/jcm12041328 36835863 PMC9967320

[31] Myska V , Genzor S, Mezina A et al Artificial-intelligence-driven algorithms for predicting response to corticosteroid treatment in patients with post-acute COVID-19. Diagnostics 2023;13:1755. 10.3390/diagnostics13101755 37238239 PMC10217330

[32] Pfaff ER , Girvin AT, Crosskey M, N3C and RECOVER Consortia et al De-black-boxing health AI: demonstrating reproducible machine learning computable phenotypes using the N3C-RECOVER Long COVID model in the All of Us data repository. J Am Med Inform Assoc 2023;30:1305–12. 37218289 10.1093/jamia/ocad077 PMC10280348

[33] Casal-Guisande M , Comesaña-Campos A, Núñez-Fernández M et al Proposal and definition of an intelligent clinical decision support system applied to the prediction of dyspnea after 12 months of an acute episode of COVID-19. Biomedicines 2024;12:854. 10.3390/biomedicines12040854 38672208 PMC11047904

[34] Jin W , Hao WEI, Shi XU et al Using multi-modal electronic health record data for the development and validation of risk prediction models for long COVID using the super learner algorithm. JCM 2023;12:7313. 10.3390/jcm12237313 38068365 PMC10707399

[35] Chow JSF , D'Souza A, Ford M et al A descriptive study of the clinical impacts on COVID-19 survivors using telemonitoring (The TeleCOVID Study). Front Med Technol 2023;5:1126258. 37020492 10.3389/fmedt.2023.1126258 PMC10067568

[36] Bartczak KT , Milkowska-Dymanowska J, Piotrowski WJ et al The utility of telemedicine in managing patients after COVID-19. Sci Rep 2022;12:21392. 36496499 10.1038/s41598-022-25348-2 PMC9736706

[37] Sivan M , Lawrence RR, O'Brien P. Digital patient reported outcome measures platform for post-COVID-19 condition and other long-term conditions: user-centered development and technical description. JMIR Hum Factors 2023;10:e48632. 37665334 10.2196/48632 PMC10592725

[38] Capecci M , Cima R, Barbini FA et al Telerehabilitation with ARC Intellicare to Cope with Motor and Respiratory Disabilities: results about the Process, Usability, and Clinical Effect of the “Ricominciare” Pilot Study. Sensors 2023;23:7238. 37631774 10.3390/s23167238 PMC10459854

[39] Koliadenko NV , Zhyvaho KS, Bursa AI. Provision of medical-psychological and psychiatric care to patients with post-Covid syndrome in telemedicine conditions. Bangladesh J Med Sci 2022;21:719–30.

[40] Estebanez-Pérez M-J , Pastora-Bernal J-M, Martín-Valero R. The effectiveness of a four-week digital physiotherapy intervention to improve functional capacity and adherence to intervention in patients with long COVID-19. IJERPH 2022;19:9566. 10.3390/ijerph19159566 35954922 PMC9367987

[41] Barnhill JL , Roth IJ, Miller VE et al Pilot observational study of patient reported outcome measures for long COVID patients in virtual integrative medical group visits. Glob Adv Integr Med Health 2023;12:27536130231174236. 10.1177/27536130231174236 37205321 PMC10186579

[42] Sarmento A , Adodo R, Hodges G et al Virtual pulmonary rehabilitation approaches in patients with post COVID syndrome: a pilot study. BMC Pulm Med 2024;24:139. 38500051 10.1186/s12890-024-02965-3 PMC10949685

[43] Rzepka-Cholasińska A , Ratajczak J, Michalski P et al Gender-related effectiveness of personalized post-COVID-19 rehabilitation. JCM 2024;13:938. 10.3390/jcm13040938 38398252 PMC10889393

[44] Kumar D , Kumar P, Ahmed I, Singh S. Integrating artificial intelligence in disease diagnosis, treatment, and formulation development: a review. Asian J Pharm Clin Res 2023;1–8. 10.22159/ajpcr.2023.v16i11.48193

[45] Patterson BK , Yogendra R, Francisco EB et al Persistence of S1 Spike Protein in CD16+ Monocytes up to 245 Days in SARS-CoV-2 Negative Post COVID-19 Vaccination Individuals with Post-Acute Sequalae of COVID-19 (PASC)-Like Symptoms. medRxiv. 2024:2024.03.24.24304286.

[46] Wang C , He T, Zhou H et al Artificial intelligence enhanced sensors—enabling technologies to next-generation healthcare and biomedical platform. Bioelectron Med 2023;9:17. 37528436 10.1186/s42234-023-00118-1 PMC10394931

[47] El-Toukhy S , Hegeman P, Zuckerman G et al A prospective natural history study of post acute sequalae of COVID-19 using digital wearables: study protocol. Res Sq 2023;rs.3.rs-3694818. 10.21203/rs.3.rs-3694818/v1

[48] Brode WM , Melamed E. A practical framework for Long COVID treatment in primary care. Life Sci 2024;354:122977. 39142509 10.1016/j.lfs.2024.122977

[49] Colton H , Neal S, Cindy Manaoat V, Sarah M. Remote patient monitoring. PSNet [internet]. 2023.

[50] Clarke-Darrington J , McDonald T, Ali P. Digital capability: an essential nursing skill for proficiency in a post-COVID-19 world. Int Nurs Rev 2023;70:291–6. 37000673 10.1111/inr.12839

[51] McIntyre RS , Greenleaf W, Bulaj G et al Digital health technologies and major depressive disorder. CNS Spectr 2023;28:662–73. 37042341 10.1017/S1092852923002225

[52] Elbéji A , Zhang L, Higa E et al Vocal biomarker predicts fatigue in people with COVID-19: results from the prospective Predi-COVID cohort study. BMJ Open 2022;12:e062463. 10.1136/bmjopen-2022-062463 PMC9684280 36414294

[53] Gentili A , Failla G, Melnyk A et al The cost-effectiveness of digital health interventions: a systematic review of the literature. Front Public Health 2022;10:787135. 36033812 10.3389/fpubh.2022.787135 PMC9403754

[54] Anawade PA , Sharma D, Gahane S. A Comprehensive review on exploring the impact of telemedicine on healthcare accessibility. Cureus 2024;16:e55996. 38618307 10.7759/cureus.55996 PMC11009553

[55] Hussein ESE , Al-Shenqiti AM, Ramadan E-S. Applications of medical digital technologies for noncommunicable diseases for follow-up during the COVID-19 pandemic. IJERPH 2022;19:12682. 10.3390/ijerph191912682 36231982 PMC9565945

[56] Tilahun B , Gashu KD, Mekonnen ZA et al Mapping the role of digital health technologies in the case detection, management, and treatment outcomes of neglected tropical diseases: a scoping review. Trop Med Health 2021;49:17. 33618757 10.1186/s41182-021-00307-1 PMC7898439

[57] Jongebloed H , Anderson K, Winter N et al The digital divide in rural and regional communities: a survey on the use of digital health technology and implications for supporting technology use. BMC Res Notes 2024;17:90. 38549176 10.1186/s13104-024-06687-x PMC10976777

[58] Facca D , Sui W. Digital health in a Broadband Land: the role of digital health literacy within rural environments. Hsi 2020;11:140–3.

[59] Petracca F , Ciani O, Cucciniello M et al Harnessing digital health technologies during and after the COVID-19 pandemic: context Matters. J Med Internet Res 2020;22:e21815. 33351777 10.2196/21815 PMC7775375

[60] Rinn R , Gao L, Schoeneich S et al Digital interventions for treating post-COVID or long-COVID symptoms: scoping review. J Med Internet Res 2023;25:e45711. 36943909 10.2196/45711 PMC10131666

[61] Dang A , Arora D, Rane P. Role of digital therapeutics and the changing future of healthcare. J Family Med Prim Care 2020;9:2207–13. 32754475 10.4103/jfmpc.jfmpc_105_20 PMC7380804

[62] Fadahunsi KP , O'Connor S, Akinlua JT et al Information quality frameworks for digital health technologies: systematic review. J Med Internet Res 2021;23:e23479. 33835034 10.2196/23479 PMC8167621

[63] Adamson B , Waskom M, Blarre A et al Approach to machine learning for extraction of real-world data variables from electronic health records. Front Pharmacol 2023;14:1180962. 37781703 10.3389/fphar.2023.1180962 PMC10541019

[64] Shah AD , Martinez C, Hemingway H. The freetext matching algorithm: a computer program to extract diagnoses and causes of death from unstructured text in electronic health records. BMC Med Inform Decis Mak 2012;12:88. 22870911 10.1186/1472-6947-12-88 PMC3483188

[65] Kovačević A , Bašaragin B, Milošević N et al De-identification of clinical free text using natural language processing: a systematic review of current approaches. Artif Intell Med 2024;151:102845. 38555848 10.1016/j.artmed.2024.102845

[66] Shih A , Choi A, Darwiche A. Compiling Bayesian network classifiers into decision graphs. Proceedings of the Thirty-Third AAAI Conference on Artificial Intelligence and Thirty-First Innovative Applications of Artificial Intelligence Conference and Ninth AAAI Symposium on Educational Advances in Artificial Intelligence; Honolulu, Hawaii, USA: AAAI Press; 2019. p. Article 977.

[67] Boumazouza R , Cheikh F, Mazure B et al A symbolic approach for counterfactual explanations. In: *14th International Conference*, SUM 2020, Bozen-Bolzano, Italy, Sep 2020, pp. 270–7, Italy: Virtual event Bozen-Bolzano, 2020.

[68] Wang KS , Yu G, Xu C et al Accurate diagnosis of colorectal cancer based on histopathology images using artificial intelligence. BMC Med 2021;19:76. 33752648 10.1186/s12916-021-01942-5 PMC7986569

[69] Chen G. Learning Symbolic Expressions via Gumbel-Max Equation Learner Network. ArXiv. 2020;abs/2012.06921.

[70] Liu Y , Yavuz S, Meng R et al HPE: answering complex questions over text by hybrid question parsing and execution. In: *Findings of the Association for Computational Linguistics: EMNLP 2023*, pp 4437–51, Singapore: Association for Computational Linguistics, 2023.

[71] Dolatabadi E , Moyano D, Bales M et al Using social media to help understand patient-reported health outcomes of post-COVID-19 condition: natural language processing approach. J Med Internet Res 2023;25:e45767. 37725432 10.2196/45767 PMC10510753

[72] Tran HTT , Lu SH, Tran HTT et al Social media insights during the COVID-19 pandemic: infodemiology study using big data. JMIR Med Inform 2021;9:e27116. 34152994 10.2196/27116 PMC8288653

[73] Simpson DB , Bird M-L, English C et al Connecting patients and therapists remotely using technology is feasible and facilitates exercise adherence after stroke. Top Stroke Rehabil 2020;27:93–102. 31762412 10.1080/10749357.2019.1690779

[74] Motahari-Nezhad H , Fgaier M, Mahdi Abid M et al Digital biomarker-based studies: scoping review of systematic reviews. JMIR mHealth uHealth 2022;10:e35722. 36279171 10.2196/35722 PMC9641516

[75] Unim B , Zile-Velika I, Pavlovska Z et al The role of digital tools and emerging devices in COVID-19 contact tracing during the first 18 months of the pandemic: a systematic review. Eur J Public Health 2024;34:i11–i28. 38946444 10.1093/eurpub/ckae039 PMC11215323

[76] Bouabida K , Lebouché B, Pomey M-P. Telehealth and COVID-19 pandemic: an overview of the telehealth use, advantages, challenges, and opportunities during COVID-19 pandemic. Healthcare 2022;10:2293. 10.3390/healthcare10112293 36421617 PMC9690761

[77] Valeriani G , Vukovic IS, Mollica R. Unconventional answers to unprecedented challenges: the Swedish experience during the COVID-19 outbreak. J Prev Med Public Health 2020;53:233–5. 32752592 10.3961/jpmph.20.235 PMC7411250

[78] Mejía-Granda CM , Fernández-Alemán JL, Carrillo-de-Gea JM et al Security vulnerabilities in healthcare: an analysis of medical devices and software. Med Biol Eng Comput 2024;62:257–73. 37789249 10.1007/s11517-023-02912-0 PMC10758361

[79] Manteghinejad A , Javanmard SH. Challenges and opportunities of digital health in a post-COVID19 world. J Res Med Sci 2021;26:11. 34084190 10.4103/jrms.JRMS_1255_20 PMC8103966

[80] Torgersen LNS , Schulz SM, Lugo RG et al Patient informed consent, ethical and legal considerations in the context of digital vulnerability with smart, cardiac implantable electronic devices. PLOS Digit Health 2024;3:e0000507. 38781144 10.1371/journal.pdig.0000507 PMC11115322

